# Pharmacokinetics of five phthalides in volatile oil of *Ligusticum sinense* Oliv.cv. *Chaxiong*, and comparison study on physicochemistry and pharmacokinetics after being formulated into solid dispersion and inclusion compound

**DOI:** 10.1186/s12906-021-03289-z

**Published:** 2021-04-22

**Authors:** Peng-yi Hu, Ying-huai Zhong, Jian-fang Feng, Dong-xun Li, Ping Deng, Wen-liu Zhang, Zhi-qiang Lei, Xue-mei Liu, Guo-song Zhang

**Affiliations:** 1 Jiangxi University of Traditional Chinese Medicine, Nanchang, 330004 China; 2 Guangxi University of Chinese Medicine, Nanning, 530200 China; 3 Nanchang Hangkong University, Nanchang, 330063 China

**Keywords:** Chaxiong, Phthalides, Solid dispersion, Inclusion compound, Physicochemical characterization, Pharmacokinetics

## Abstract

**Backgrounds:**

The dried rhizome of *Ligusticum sinense* Oliv.cv. *Chaxiong* has been used to treat cardiovascular and cerebrovascular diseases, atherosclerosis, anemia and stroke. A high purity extract from chaxiong (VOC, brownish yellow oil) was extracted and separated. Its main components were senkyunolide A (SA, 33.81%), N-butylphthalide (NBP, 1.38%), Neocnidilide (NOL, 16.53%), Z-ligustilide (ZL, 38.36%), and butenyl phthalide (BP, 2.48%), respectively. Little is known about the pharmacokinetics of these phthalides in Chaxiong, and different preparations to improve the physicochemistry and pharmacokinetics of VOC have not been investigated.

**Methods:**

At different predetermined time points after oral administration or intravenous administration, the concentrations of SA, NBP, NOL, ZL and BP in the rat plasma were determined using LC-MS/MS, and the main PK parameters were investigated. VOC-P188 solid dispersion and VOC-β-CD inclusion compound were prepared by melting solvent method and grinding method, respectively. Moreover, the physicochemical properties, dissolution and pharmacokinetics of VOC-P188 solid dispersion and VOC-β-CD inclusion compound in rats were assessed in comparison to VOC.

**Results:**

The absorptions of SA, NBP, NOL, ZL and BP in VOC were rapid after oral administration, and the absolute bioavailability was less than 25%. After the two preparations were prepared, dissolution rate was improved at pH 5.8 phosphate buffer solution. Comparing VOC and physical mixture with the solid dispersion and inclusion compound, it was observed differences occurred in the chemical composition, thermal stability, and morphology. Both VOC-P188 solid dispersion and VOC-β-CD inclusion compound had a significantly higher AUC and longer MRT in comparison with VOC.

**Conclusion:**

SA, NBP, NOL, ZL and BP in VOC from chaxiong possessed poor absolute oral bioavailability. Both VOC-P188 solid dispersion and VOC-β-CD inclusion compound could be prospective means for improving oral bioavailability of SA, NBP, NOL, ZL and BP in VOC.

## Background

Stroke, a common neurological disease with a high risk of suddenness, disability or death, is mainly caused by atherosclerosis, vasospasm, cerebral ischemia and hypoxia. It is the second largest cause of death in the world [26, 27]. It impairs capabilities of communication, cognition, and motor function in patients, so it substantially affects the quality of life of stroke patients [2]. The dried rhizome of *Ligusticum sinense* Oliv.cv. *Chaxiong* has been used to treat cardiovascular and cerebrovascular diseases, atherosclerosis, anemia and stroke [35, 41]. Chaxiong contains monomeric phthalides, dimer phthalides, organic acid, phenols, flavonoids, coumarins, alkaloids, volatile oils and polysaccharides, among which phthalides are the main chemical components. Many of the naturally occurring phthalides display different biological activities including antibacterial, antifungal, insecticidal, cytotoxic, and anti-inflammatory effects, among many others [15]. Some monomeric phthalides have shown their ability to attenuate certain neurological diseases, including stroke, Alzheimer’s and Parkinson’s diseases [15]. Some new phthalides and some known phthalides in chaxiong were extracted and separated, and had been shown to have a protective effects against neuronal impairment induced by deprival of oxygen and glucose [35, 36].

**Fig. 1:**
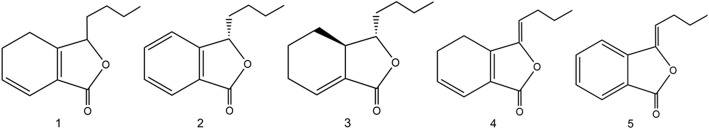
The chemical structures of 5 active phthalides in VOC. 1. senkyunolide A (SA), 2. N-butylphthalide (NBP), 3. Neocnidilide (NOL), 4. Z-ligustilide (ZL), 5. butenyl phthalide (BP)

At present, a high purity extract from chaxiong (VOC, brownish yellow oil) was extracted and separated in our labs. Its main components were phthalides, and the contents were senkyunolide A (SA, 33.81%), N-butylphthalide (NBP, 1.38%), Neocnidilide (NOL, 16.53%), Z-ligustilide (ZL, 38.36%), and butenyl phthalide (BP, 2.48%), respectively [49]. The chemical structures of these compounds are known as monomeric phthalides and are shown in Fig. 1. Most of these five phthalides have been reported to have therapeutic effects on stroke in vivo or in vitro.

ZL provided the potent neuroprotective effects against hemorrhagic stroke by inhibiting Prx1/TLR4/NF-kB signaling, the subsequent immune and neuroinflammation lesions [12]. In addition, ZL also attenuated ischemia reperfusion-induced hippocampal neuronal apoptosis via activating the PI3K/Akt pathway [37]. NBP is a cardiovascular drug currently used for the treatment of cerebral ischemia, and was approved for marketing in 2002 by the State Food and Drug Administration (SFDA) of China in the form of soft capsule and infusion drip [5, 6]. Many clinical studies indicated that NBP could improve the symptoms of ischemic stroke and benefit to the long-term recovery. The potential mechanisms of NBP may attribute to anti-oxidant, anti-inflammation, anti-apoptosis, anti-thrombosis, protection of mitochondria and so on [34]. SA could inhibit the production of proinflammatory mediators in lipopolysaccharide (LPS)-stimulated murine BV-2 microglial cells and human peripheral blood monocyte derived macrophages, and it may be considered as potential complementary drug candidates for treating inflammatory processes associated with cerebrovascular diseases [23]. Few studies have been reported about NOL and BP in the treatment of stroke.

Pharmacokinetic studies play an increasingly important role in drug discovery and development processes, a poor pharmacokinetic profile due to lower bioavailability and rapid clearance limits drug efficacy [24]. NBP underwent extensive metabolism after an oral administration of 200 mg NBP and 23 metabolites were identified in human plasma and urine [5, 6]. Oral bioavailability of LIG was low (2.6%), and seven metabolites of LIG were unequivocally characterized as NBP, senkyunolide I and senkyunolide H [40]. The pharmacokinetic parameters of SA, NBP and ZL showed significant differences between Rhizoma Chuanxiong group and the combination of Radix Angelicae Dahuricae and Rhizoma Chuanxiong group (*P* < 0.05) [32, 33]. The i.v. clearance (CL) of ZL after Chuanxiong extract administration was significantly higher than that dosed in its pure form, suggesting significant interaction between ZL and components present in the extract [40]. Until now, the pharmacokinetics of SA, NBP, NOL, ZL and BP in VOC of chaxiong is not yet known, it is of great significance to study the pharmacokinetic parameters of these five phthalides in vivo for ensuring their efficacy and dosage form design. Therefore, the objective of this study was to investigate the pharmacokinetics of SA, NBP, NOL, ZL and BP after oral and intravenous administration of VOC.

Preliminary studies and pharmacokinetic experiments showed that VOC had poor water solubility and instability, and the bioavailability of each active ingredient was no more than 25% in rats, and therefore, VOC was prepared into solid dispersions and inclusion compounds to improve that [9, 46]. Solid dispersion was an established concept for drug solubility and bioavailability enhancement but strongly dependent on the choice of an appropriate carrier and preparation technique [3, 31]. Poloxamer 188 (P188) is a kind of non-ionic surfactant approved by FDA, commonly used with insoluble drugs as solubilizer and surfactant, based on high drug loading, low melting point, hydrophilicity and safety [7]. On the other hand, in the pharmaceutics to increase water solubility and physicochemical stability, cyclodextrins are used in drug: cyclodextrin inclusion compound [4, 10]. β-Cyclodextrins (β-CD) have also been considered to be used in delivery systems based on their ability to form inclusion compounds and improve the solubility and bioavailability of drugs [25].

In this work, VOC-P188 solid dispersion and VOC-β-CD inclusion compound were prepared by melting solvent method and grinding method, respectively. The physicochemical properties of the formulations were investigated using SEM (scanning electron microscopy), DSC (differential scanning calorimetry), PXRD (powder X-ray diffraction) and FT-IR (Fourier Transform Infrared Spectrometer). Furthermore, studies of the dissolution in vitro and pharmacokinetics in rats were carried out in comparison with VOC. In view of the irreplaceable role of animals in pharmacokinetic studies, this study used Sprague Dawley rats.

## Methods

### Materials and reagents

SA (purity> 98%), NBP (purity> 98%), ZL (purity> 98%) and dehydrocostus lactone (DL, internal standard, purity> 99%) were purchased from Desite Biotech Co., Ltd., Chengdu, China. NOL (purity> 98%) and BP (purity> 98%) were purchased from Chroma Biotechnology Co., Ltd., Chengdu, China.

β-cyclodextrin (β-CD) was purchased from Damao Chemical Reagent Co., Ltd., Tianjin, China. Poloxamer 188 (P188) was purchased from BASF Co., Ltd. Potassium bromide (SP grade, purchased from Maclean Biochemical Technology Co., Ltd. Shanghai, China. Tween 80 was purchased from KaiTong Chemical Reagent Co., Ltd. Tianjin, China. Methanol and acetonitrile are HPLC grade, purchased from Thermo Fisher.

### Animals

Male Sprague Dawley rats(weight: 220 ± 20 g) were purchased from Silaikejingda Laboratory animals Co., Ltd., Hunan, China, the number of animal Quality License: SCXK 2016–0002. The animals were maintained in a room with a constant temperature of 23 ± 1 °C; A relative humidity of 30–40%; light for 12 h from 06:00 to 18:00; and ad libitum food and purified water. The studies were approved by the animal ethics committee of Jiangxi University of Traditional Chinese Medicine (jzllsc0137). All animals were maintained in accordance with the guidelines outlined by the Chinese legislation on the ethical use and care of laboratory animals. All efforts were made to minimize both animal suffering and the number of animals used to produce reliable data.

### Extraction process of VOC

The crushed air-dried aerial parts of *Ligusticum sinense* Oliv.cv. *Chaxiong* were extracted two times with 95% ethanol, each for 1.5 h. After filtration and removal of the solvent under reduced pressure, the residue was partitioned into H_2_O and extracted with EtOAc. The EtOAc fraction was separated by Pope Molecular Distillation after solvent was recovered, for the light component and heavy component. The light component was VOC (a brownish yellow oil, 0.93% of yield, w/w) [17].

### HPLC analysis

The constituents of VOC were identified by high performance liquid chromatography (HPLC), which had been published in *Chinese Traditional Patent Medicine* [49]. The high performance liquid chromatographic system consisted of a LC-20AT pump (Shimadzu, Kyoto, Japan), a SIL-20A injector with 20 μl loop (Shimadzu, Kyoto, Japan), a SPD-M20A PAD–visible detector (Shimadzu, Kyoto, Japan) and a LC solution workstation. Separation was performed with a Kromasil 100–5-C18 column (250 mm × 4.60 mm; 5 μm) from AkzoNobel Co. (Sweden). The mobile phase was water as mobile phase A and acetonitrile as mobile phase B. The linear gradient elution program was set according to preliminary tests: 38–42% B, 0–10 min; 42–45% B, 10–36 min; 45–48% B, 36–55 min; 48–38% B, 55–60 min, with a flow rate of 1.0 ml/min. The detector was set at 230 nm for NOL, NBP and BP, and 280 nm for SA and ZL, and all the measurements were performed at 30 °C. The injection volume was set at 20 μl.

### Preparation of solid dispersion and inclusion compound of VOC

Solid dispersion of VOC was prepared by using the solvent melting method. Briefly, VOC was dissolved in minimum volume of absolute alcohol, and P188 at drug to carrier ratios (1:6, w/w) was melted at 60 °C under water bath, then the VOC solution was added to P188 to obtain a homogeneous mixture. The solvent was removed under vacuum in a rotary evaporator at 40 °C and 45 rpm for 2 h, and the resulting dispersion was kept in refrigerator at − 80 °C. After 4 h, the dispersion was crushed and sieved through 40-mesh, and stored in a desiccator at room temperature for use [32, 33].

The preparation of the β-CD-VOC was carried out by co-dissolution. A solution of β-CD in three times purified water (v/w), was added a solution of VOC in minimum volume of absolute alcohol at drug to carrier ratios (1:8, w/w), and the resulting solution was allowed to stir for 1.5 h. The mixture was then filtered, washed by a little water and an appropriate amount of ether for three times to remove the unencapsulated VOC in the outer layer, and dried at 40 °C for 6 h. Finally, the inclusion compound was crushed and sieved through 65-mesh, and stored in a desiccator at room temperature for use [1].

### In vitro dissolution studies

The results of our previous study showed that the apparent permeability coefficient of SA, NBP, NOL, ZL and BP in duodenum, jejunum, ileum and colon of rats were greater than 2.0 × 10^− 6^ cm/s by the everted intestinal sac model. The cumulative absorption of the five components in VOC was duodenal > colon > ileum > jejunum, which indicated that the small intestine was the main absorption site of the drug, so pH 5.8 phosphate buffer solutions was selected as its dissolution medium. Dissolution studies were performed according to the USP XXI method 2 (paddle method) at 37 ± 0.5 °C at 50 rpm. 900 ml of dissolution medium were used as standard volume. Dissolution samples (1 mL) were withdrawn at the specified times of 15, 30, 45, 60, 90, 120 min from Dissolution cups, and 1 ml of dissolution medium was replenished in the same time. Samples were filtered through a 0.45 μm nylon disc filter and were measured absorbance at 230 nm by HPLC [11].

### Physicochemical characterization

#### Morphology

Their morphological aspects were assessed using the Quanta250 scanning electron microscope (FEI Company, USA). The samples were put on a brass disc by double-sided adhesive carbon tape. Employing an EMI Teck Ion Sputter (K575K), they were sputter-coated with platinum under vacuum (8 × 10^− 3^ mbar) for 4 min at a current of 15 mA and 100% turbo speed.

#### Thermal features

The thermal analysis of solid dispersion and inclusion compound of VOC were determined using DSC 8000 differential scanning calorimeter (PerkinElmer, America). Sample was placed in alumina crucible and scanned at a 10 °C/min linear rate from − 40 °C to 300 °C in an atmosphere of nitrogen.

#### Structural aspects

Their crystallinity was determined utilizing a powder X-ray diffractometer (PXRD) (D8 ADVANCE X-ray diffractometer, Rigaku Co.; Bruker CO., Germany). The analysis was carried out at 25 °C using a Cu Kα1 monochromatic radiation source at a current of 40 mA and a voltage of 40 kV. PXRD patterns were obtained in the range of 4–50° with a 2θ scanning mode, a rate meter with time constant of 0.02 pulse/sec and a scan speed of 5°/min [8].

The FT-IR spectra of the samples were recorded on a Sprucm two spectrometer (PerkinElmer, USA) using the (KBr) disc technique. For FT-IR measurements, all the samples were mixed with KBr separately in a clean glass mortar and compressed to obtain a thin tablet. Background spectra were obtained with a KBr tablet for each sample. The wave number range is 4000 ~ 400 cm^− 1^, and the resolution is 4 cm^− 1^.

### Pharmacokinetic research

#### Animal treatment

The intravenous injection solution was prepared by dissolving 0.15 g of VOC in 25 ml water containing 3 ml 10% Tween-80 as solubilizer. The oral solution was prepared by dissolving 0.35 g of VOC in 25 ml normal saline containing 3 ml 10% Tween-80 as solubilizer. The VOC-P188 solution was prepared by mixing VOC-P188 5 g in 25 ml water with magnetic stirring apparatus. The VOC-β-CD solution was prepared by mixing VOC-β-CD 3.5 g in 25 ml water with magnetic stirring apparatus.

Twenty-four male SD rats (220 ~ 250 g) were randomly divided into four groups, 6 in each group. After fasting for 12 h before administration, the rats in group 1 were given VOC by tail vein injection at a dose of 30 mg/Kg, the rats at second group were oral administration of VOC solution, the dosage was 120 mg/Kg based on the mass of VOC. The rats at groups 3 and 4 were given VOC-P188 and VOC-β-CD, respectively. The dosage of each active component was shown in Table 1 below.

**Table 1:** Specific dosage of each index component in injection group and oral administration group (mg/kg)

Group	SA	NBP	NOL	ZL	BP
1. VOC(iv)	10.51	0.40	5.05	11.63	0.61
2. VOC(po)	42.04	1.62	20.21	46.51	2.45
3.VOC-P188	42.40	1.65	20.16	45.28	2.45
4.VOC-β-CD	43.89	1.72	19.80	36.30	2.03

Blood samples (0.3 ml) were collected from the eyes at 0.08, 0.17, 0.33, 0.5, 1, 1.5, 2, 4, 6, 8, 12 and 24 h after administration, and they were immediately transferred into a heparinized Eppendorf (EP) tube. The blood samples were then centrifuged at 13,000 r/min for 10 min. The plasma samples were stored at − 80 °C for later analysis [16, 21].

The rats were anaesthetised with an intraperitoneal injection of 10% pentobarbital sodium (4 ml/kg) and subsequently sacrificed by rapid decapitation after experimentation.

#### Blood sample preparation

The thawed plasma sample (0.1 ml) was transferred to another EP tube and 20 μl of DL, 50 μl of 2% formic acid and 500 μl of methanol were added. The mixture was vortexed for 2 min, and centrifuged at 13000 r/min for 10 min. The supernatant was transferred into a clean EP tube, and evaporated to dryness under a stream of nitrogen at 40 °C. The residue was dissolved in 100 μl of methanol. The supernatant was injected into the HPLC system [14].

#### LC-MS/MS measurement

An HPLC system consisting of a solvent-delivery system LC-30 AD, an autosampler SIL-30 AC, a column oven CTO-30 AC, a solvent degasser DGU-20A3, and a controller CBM-20A from AB Sciex (Framingham, MA, USA) was used in the study. Separation was conducted using a Shim-pack GIST C18-AQ HP column (2.1 mm × 100 mm, 3 μm). The column oven was maintained at 35 °C. The mobile phase was 0.1% formic acid in water as mobile phase A and acetonitrile as mobile phase B. The linear gradient elution program was set according to preliminary tests: 45–60% B, 0–2 min; 60–65% B, 2–6 min; 65–95% B, 6–7 min; 95–45% B, 7–9 min and 45% B, 9–10 min, with the flow rate kept at 0.3 ml/min. The injection volume was set at 2 μl.

The MS analysis was performed on a 4500 QTRAP™ system from Applied Biosystems (AB Sciex) equipped with Turbo V sources and Turbo Ion Spray™ (TIS) interface. Electrospray ionization was performed in positive mode. Ion source: electrospray ion source (ESI source); detection method: multiple reaction monitoring (MRM); ion source temperature: 500 °C; desolvent gas temperature: 550 °C; desolvent gas flow rate: 800 l/h. The detection ion pair, retention time, declustering voltage and collision energy of each index component and internal standard were shown in Table 2.

**Table 2:** Conditions of mass spectrum on each index component and internal standard

component	[M + H]^+^ (m_1_/m_2_)	Declustering voltage(U/V)	Collision energy(E/eV)	retention time(t/min)
SA	193.1 → 136.8	76	17	4.17
NBP	191.3 → 116.9	78	30	4.37
NOL	195.0 → 149.0	79	14	5.09
ZL	191.1 → 173.1	88	40	5.39
BP	189.1 → 128.0	76	35	5.52
DL(IS)	231.0 → 185.1	78	12	5.85

#### Preparation of stock, calibration standards and quality control samples

The standard stock solution was prepared by dissolving 34.13 mg of SA, 10.41 mg of NBP, 36.56 mg of NOL, 5.11 mg of ZL and 11.39 mg of BP in 25 ml methanol. The mixed stock solutions were diluted in methanol to prepare a series of standard working solutions. All the stock solutions were kept at 4 °C before use. An IS solution of DL was diluted with methanol to yield a final concentration of 3.38 μg/ml. Calibration samples were prepared by spiking 100 μl standard working solutions into 100 μl blank rat plasma at the following concentrations: 84.33, 168.67, 337.34, 647.68 and 5397.40 ng/ml for SA, 5.12, 10.25, 20.50, 41.00 and 327.92 ng/mL for NBP, 25.16, 50.32, 100.65, 201.30 and 1610.40 ng/ml for NOL, 90.42, 180.84, 361.69, 723.38 and 5787.01 ng/ml for ZL and 5.70, 11.39, 22.78, 45.55 and 364.48 ng/ml for BP. Quality control (QC) samples including 168.67, 337.34 and 647.68 ng/ml for SA, 10.25, 20.50 and 41.00 ng/ml for NBP, 50.32, 100.65 and 201.30 ng/ml for NOL, 180.84, 361.69 and 723.38 ng/ml for ZL and 11.39, 22.78 and 45.55 ng/ml for BP were prepared in a similar way to the calibration standards.

#### Assay validation

An aliquot of 20.0 μl IS (DL, 3.38 μg/ml) was pipetted into 100 μl of the plasma sample. After that, 50 μl of 2% formic acid and 500 μl of methanol were added. The mixture was vortexed for 2 min, and centrifuged at 13000 r/min for 10 min. The supernatant was transferred into a clean EP tube, and evaporated to dryness under a stream of nitrogen at 40 °C. The residue was dissolved in 100 μl of methanol. The supernatant was injected into the HPLC system. The method was verified according to the following criteria: specificity, linearity, accuracy, precision, extraction recovery, matrix effect and stability. Precision and accuracy were carried out by quantifying QC samples at low, medium and high concentrations in six replicates on a single day (intra-day) and on five successive validation days (inter-day). The extraction recoveries were evaluated by comparing the peak areas obtained from extracted spiked samples (low, medium, high concentration) with those from post-extraction blank matrix spiked with the appropriate analytes. The matrix effect was investigated by comparing the peak areas obtained from the extracted matrix spiked with standard solutions with the corresponding standard solutions. The stability of the analytes in rat plasma was investigated by analyzing QC samples under three different storage conditions: 12 h at room temperature, three freeze (− 80 °C) and thaw (room temperature) cycles, and keeping at 4 °C for 24 h. All stability studies utilizing QC samples were determined using a calibration curve of freshly prepared solutions.

#### Pharmacokinetic analysis

The plasma SA, NBP, NOL, ZL and BP concentrations were evaluated using the equation from the standard curves that were run with each batch of samples. The plasma concentration-time data were analyzed with the Drug and Statistics 2.0 software package to determine the pharmacokinetic parameters. The observed Cmax value was obtained from the observed data and the observed AUC_0 → t_ value was calculated using the trapezoidal rule. The absolute bioavailability of VOC and the relative bioavailability of VOC-P188 and VOC-β-CD were calculated, the formula for calculating absolute bioavailability (*F*_1_) and relative bioavailability (*F*_2_):

$$ {F}_1=\left({\mathrm{AUC}}_{\mathrm{po}}\times {\mathrm{D}}_{\mathrm{iv}}\right)/\left({\mathrm{AUC}}_{\mathrm{iv}}\times {\mathrm{D}}_{\mathrm{po}}\right)\times 100\% $$

$$ {F}_2=\left({\mathrm{AUC}}_{\mathrm{R}}\times {\mathrm{D}}_{\mathrm{T}}\right)/\left({\mathrm{AUC}}_{\mathrm{T}}\times {\mathrm{D}}_{\mathrm{R}}\right)\times 100\% $$

Where AUC is the area under the temporal curve of blood drug concentration (μg/l*h), D is administration dosage (mg/kg); the subscripts iv and po are injection and oral administration, respectively; the subscript R is reference drug (VOC); the subscript T is solid dispersion or inclusion compound.

#### Statistical analysis

The results were expressed as the mean ± SD. The differences in the pharmacokinetic parameters among the four groups were tested using one-way analysis of variance (ANOVA), followed by the Fisher’s least significant difference (LSD) test to analyze differences among multiple groups (SPSS19.0 Statistical software). Values of *p* < 0.05 were considered statistically significant.

## Results

### The composition of VOC

**Fig. 2:**
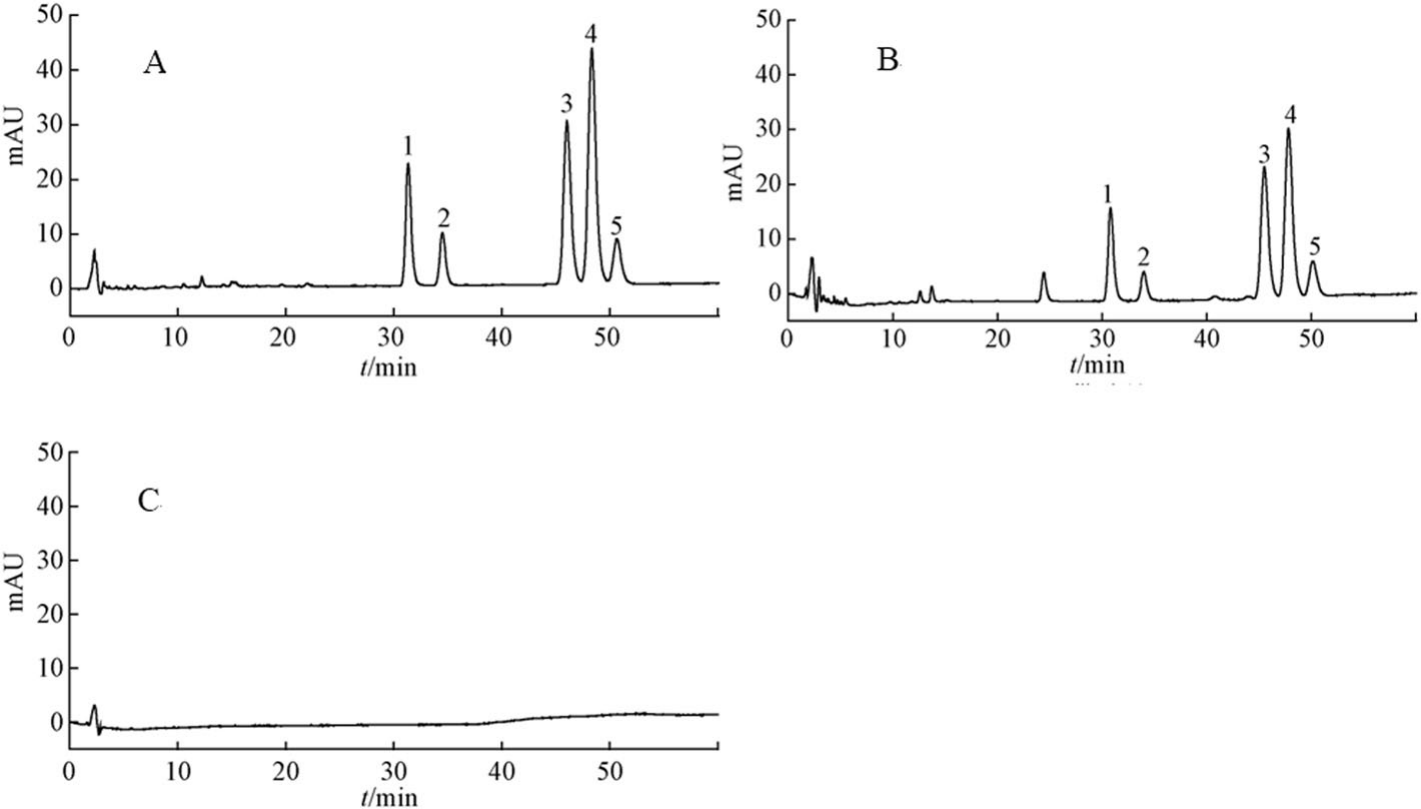
HPLC chromatograms of various constituents; **a** reference substances; **b** sample; **c** negative sample. 1. senkyunolide A (SA); 2. N-butylphthalide (NBP) 3. Neocnidilide (NOL); 4. Z-ligustilide (ZL); 5. butenyl phthalide (BP)

Typical chromatograms obtained for VOC samples were shown in Fig. 2. The retention time of SA, NBP, NOL, ZL and BP were 31.55, 34.16, 46.14, 48.43 and 50.76 min, respectively. There was no interference peak occurring close to that of 5 phthalides. Resolution between adjacent main peaks was greater than 1.5. The most abundant compounds included ZL (38.36%), SA (33.81%), NOL (16.53%), BP (2.48%) and NBP (1.38%), which presented about 93.56% of VOC.

### Dissolution of VOC-P188 and VOC-β-CD in vitro

**Fig. 3:**
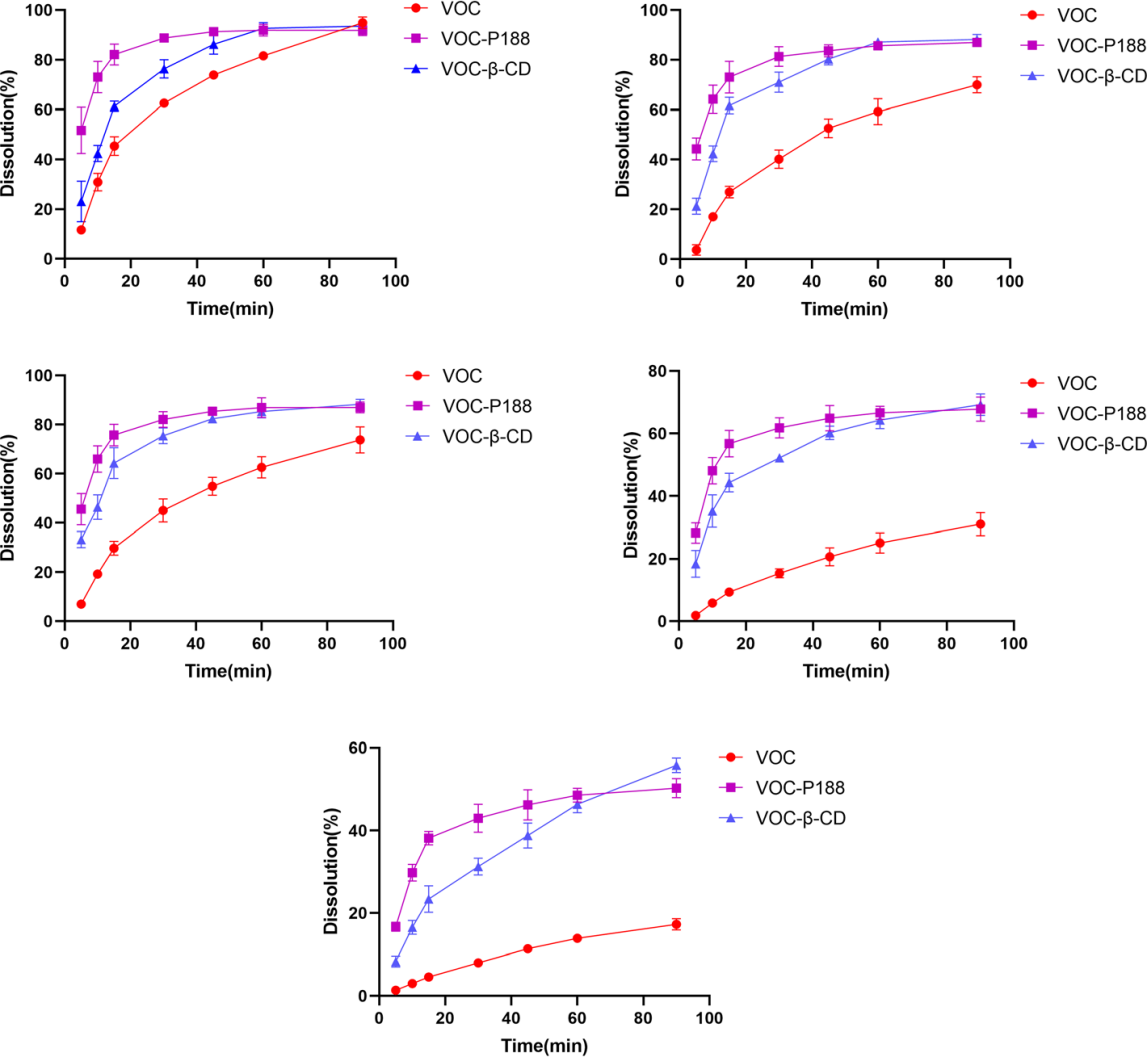
Dissolution curve of VOC, VOC-P188 and VOC-β-CD in pH 5.8 PBS

Dissolution curve of SA, NBP, ZOL, ZL and BP in VOC, VOC-P188 and VOC-β-CD in pH 5.8 PBS in vitro were shown in Fig. 3. The dissolution rate of five active components was significantly improved in vitro after VOC was prepared into two new formulations. Among them, the dissolution rate of the ZL and BP was relatively slow, and the dissolution platforms at 90 min were low, because they had poor solubility in pH 5.8 PBS. In this study, P188 had a more remarkable improvement on the dissolution rate of the five bioactive ingredients of VOC than β-CD.

### Physicochemical characterization

**Fig. 4:**
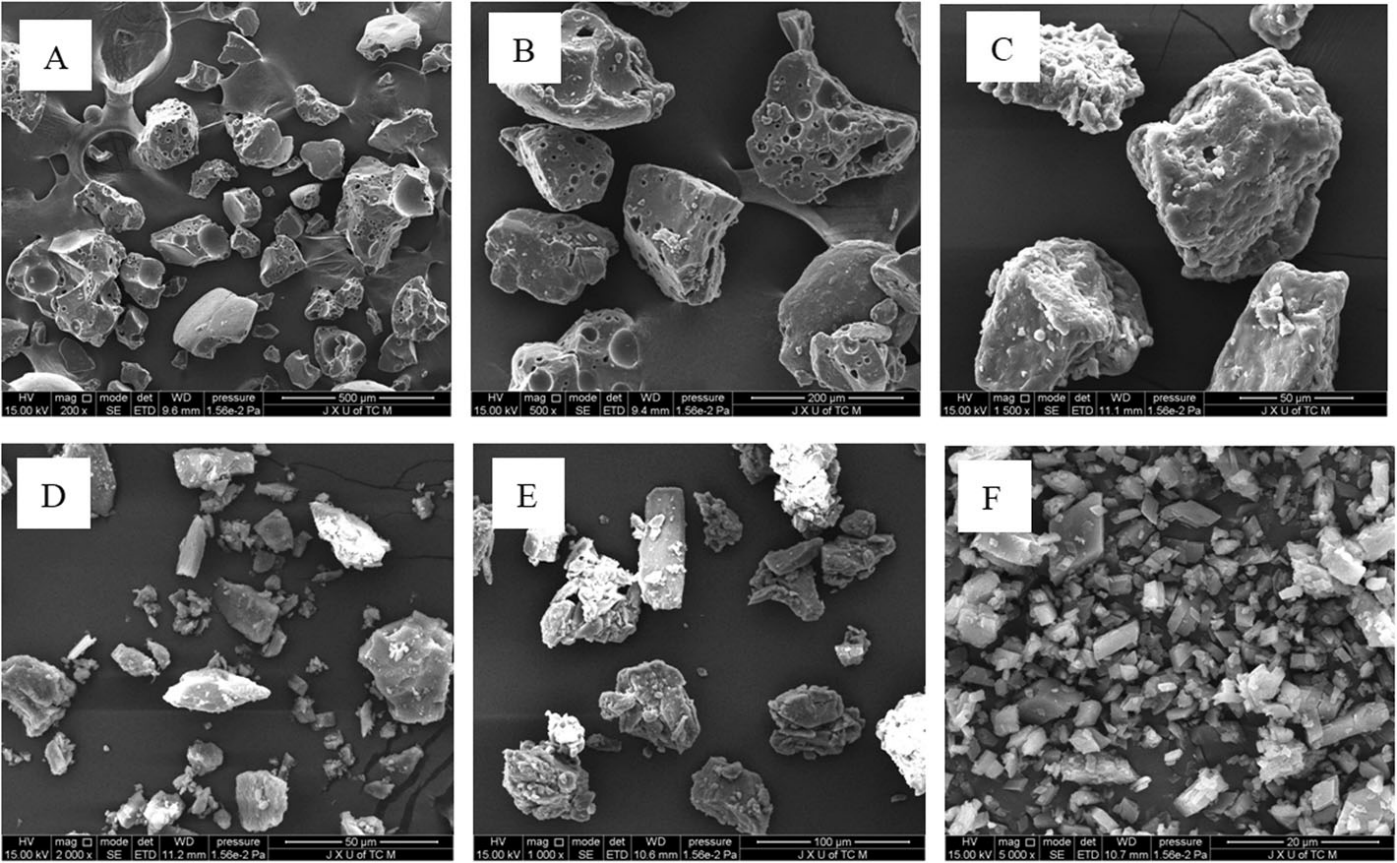
SEM graphs of solid dispersion, inclusion compound of VOC, carrier and physical mixture (× 2000). **a** P188; **b** physical mixture of VOC and P188; **c** VOC-P188; **d** β-CD; **e** physical mixture of VOC and β-CD; **f** VOC-β-CD

The SEM images of VOC-P188, VOC-β-CD, carrier and their physical mixture were shown in Fig. 4. P188 mainly existed in irregular shapes such as squares and diamonds with a particle size of about 50 ~ 100 μm. After physical mixing with VOC, there was not much change in morphology. The morphology of Fig. 4c was obviously different from A and B, It mainly existed in the shape of square and sphere, and the particle size became smaller (about 20-40 μm) and more asperous when compared with A and B, indicating that VOC and P188 formed a solid dispersion, mainly in an amorphous state.

β-CD (Fig. 4d) mainly existed in irregular crystal shapes such as diamonds and squares, with particle size of 3-20 μm. There was litter change in the particle size and morphology after mixing with VOC. VOC-β-CD (Fig. 4f) was more smooth when compared with D and E, which existed in cube or diamond shape, with the particle size of 2 ~ 6 μm, indicating that the drug molecule had been included in the cavities of β-CD.

**Fig. 5:**
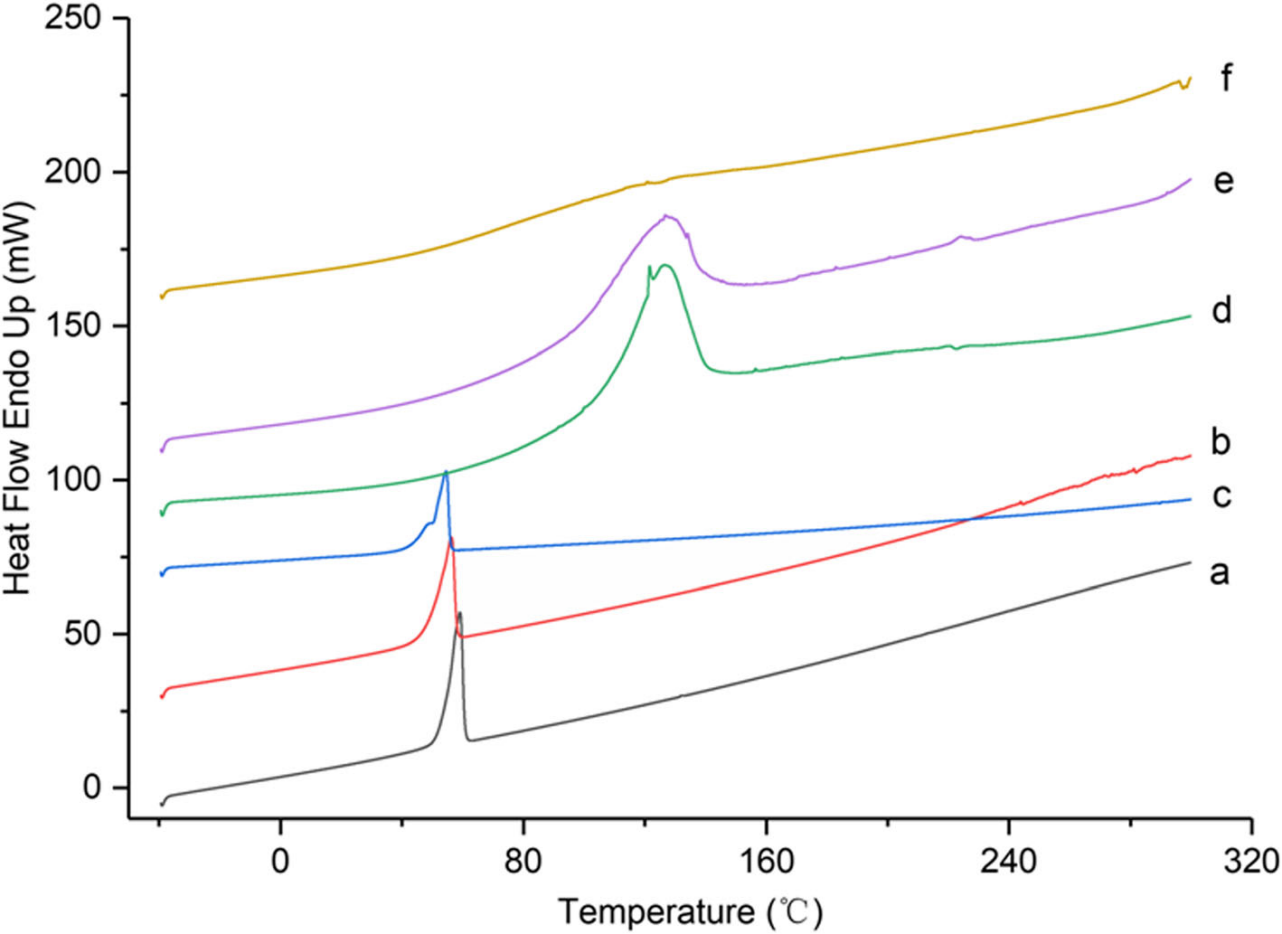
DSC spectra of VOC solid dispersion, inclusion compound, carrier and physical mixture. **a** P188; **b** physical mixture of VOC and P188; **c** VOC-P188; **d** β-CD; **e** physical mixture of VOC and β-CD; **f** VOC-β-CD

The DSC spectra of VOC-P188, VOC-β-CD, carrier and its physical mixture were shown in Fig. 5. P188 carrier (Fig. 5a) had a strong absorption peak of P188 (melting point of P188, 57 ~ 60°C ) at 59.07°C. The absorption peak of physical mixture of VOC and P188 was slightly weakened. The absorption peak of VOC-P188 was earlier (Pea = 54.54 °C), the intensity was significantly weakened, and the shoulder peak appeared. VOC had lower melting point and boiling point so that the absorption peak was earlier. The appearance of a shoulder peak may be explained that VOC melted first and P188 melted later during the temperature programming process. Compared with figure a and b, it showed that a new phase occurred. The absorption peak of β-CD was blunt and broad at 126.50°C, the absorption peak of the physical mixture of VOC and β-CD had weakened, and the absorption peak of the VOC-β-CD even disappeared, which indicated that VOC had been encapsulated in the cavities of β-CD [20].

**Fig. 6:**
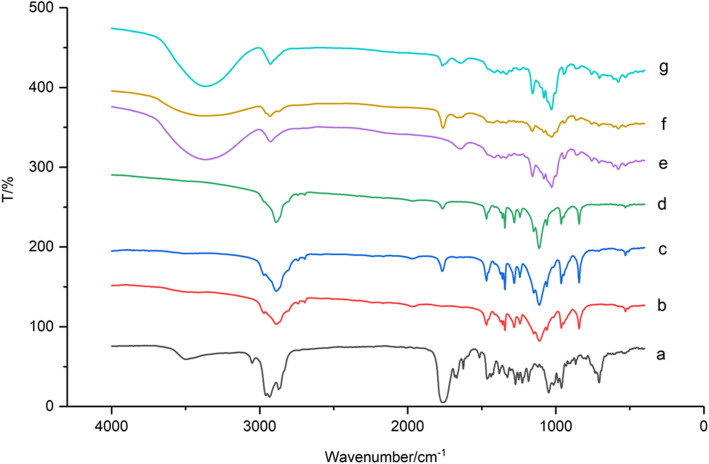
Infrared spectrum of VOC solid dispersion, inclusion compound, carrier and physical mixture. **a** VOC; **b** P188; **c** physical mixture of VOC and P188; **d** VOC-P188; **e** β-CD; **f** physical mixture of VOC and β-CD; **g** VOC-β-CD

The infrared spectrum of VOC-P188, VOC-β-CD, VOC, carrier and their physical mixture were shown in Fig. 6. VOC had a strong C-H plane at 2960 cm^− 1^ and a strong C = O stretching vibration at 1756 cm^− 1^. Combining with the VOC molecular structure formula, the absorption peak at 1650 ~ 1800 cm^− 1^ was the characteristic absorption peak of its functional group. P188 had C-H plane of stretching vibration at 2850 cm^− 1^, the characteristic absorption peak of the physical mixture of VOC and P188 had weakened at 1750 cm^− 1^, and the characteristic absorption of solid dispersion of VOC-P188 at 1750 cm^− 1^ peak almost disappeared completely, and the absorption peak at 900 ~ 1400 cm^− 1^ was also greatly reduced, indicating that VOC and P188 had formed a solid dispersion, and the drug molecules mainly existed in amorphous form [22]. Figure 6e and g were the infrared spectrum of β-CD, physical mixtures of β-CD and VOC, VOC-β-CD. It can be seen from the figure that these three substances had relatively few absorption peaks, The physical mixture of β-CD and VOC had a strong characteristic absorption peak (C = O) at 1762 cm^− 1^, but the characteristic absorption peak of VOC-β-CD almost disappeared, indicating that the drug molecule was included in the cavity, inclusion compounds had been formed.

**Fig. 7:**
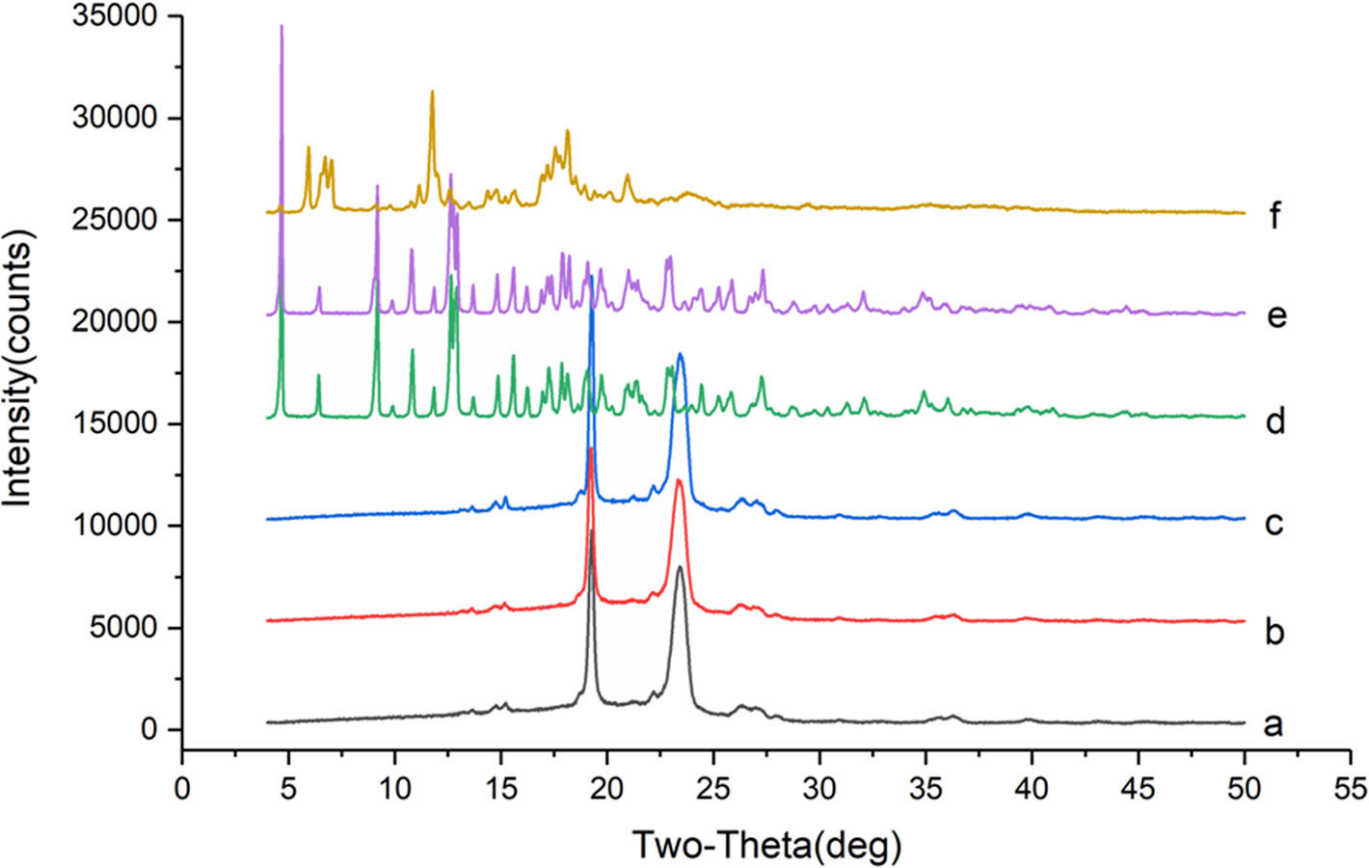
PXRD spectrum of solid dispersion, inclusion compound, carrier and physical mixture. **a** P188; **b** physical mixture of VOC and P188; **c** VOC-P188; **d** β-CD; **e** physical mixture of VOC and β-CD; **f** VOC-β-CD

The PXRD spectrum of VOC-F188, VOC-β-CD, carrier and their physical mixture were shown in Fig. 7. The PXRD spectrum of P188, the physical mixture of VOC and P188 were no significant difference in peak shape because VOC was a liquid that had no diffraction peak. Therefore, the peak shape of the physical mixture was basically the same as that of the blank excipient. However, the peak intensity of VOC-P188 had been enhanced, indicating that there was a new phase in the sample. The PXRD spectrum of β-CD (Fig. 7d) had strong and dense diffraction peaks at 2θ = 15 ~ 20°, the intensity of the peak of the physical mixture with VOC was weakened, and the diffraction peak of VOC-β-CD was greatly reduced, indicating the formation of new phases.

### Method validation

There were no interference peak close to that of SA, NBP, NOL, ZL and BP in the blank plasma. The standard curves of the analytes all exhibited good linearity within the described ranges for each analyte. Linear ranges, regression equations, lower limit of quantification (LLOQs) and correlation coefficients obtained from typical calibration curves were listed in Table 3. The intra- and inter-day precisions were in the ranges 3.88–8.15 and 3.60–10.32%, respectively. The accuracy of the analytes was within ±15%. The recovery of the analytes was shown in Table 4. Under these three conditions, the actual concentration of each index component accounted for 80% ~ 92% of the theoretical concentration, and the RSD was not more than 10%. The results showed that the analytes were stable in rat plasma under the above conditions. These data indicated that the analytical method was specific and sensitive, and could be used to determine SA, NBP, NOL, ZL and BP in rat plasma accurately.

**Table 3:** Regression equations, linear ranges and LLOQs of the analytes in plasma

Analyte	Linear range (ng/ml)	Regression equation	r	LLOQ
SA	84.33 ~ 5397.40	y = 2.4 × 10^−3^x-1.2 × 10^− 1^	0.9972	84.33
NBP	5.12 ~ 327.92	y = 1.0 × 10^−3^x-1.1 × 10^− 3^	0.9996	5.12
NOL	25.16 ~ 1610.40	y = 3.6 × 10^−3^x-8.9 × 10^− 2^	0.9940	25.16
ZL	90.42 ~ 5787.01	y = 1.0 × 10^−5^x-5.0 × 10^−5^	0.9960	90.42
BP	5.70 ~ 364.48	y = 2.5 × 10^−3^x-3.3 × 10^−2^	0.9953	5.70

**Table 4:** Summary of accuracy, precision and recovery for the analytes in rat plasma. Values represent the means ± standard error (*n* = 6)

Analyte	Concentration (ng/ml)	Intra-day		Inter-day		Recovery (%, mean ± SD)
		Accuracy (%)	RSD (%)	Accuracy (%)	RSD (%)	
SA	168.67	86.23 ± 3.62	3.89	85.78 ± 5.89	8.37	82.14 ± 4.21
	337.34	89.33 ± 6.55	7.01	86.87 ± 6.21	7.32	84.36 ± 4.87
	647.68	92.10 ± 4.10	5.89	89.24 ± 3.39	3.60	85.01 ± 5.87
NBP	10.25	88.11 ± 5.88	6.21	85.05 ± 7.21	7.44	89.02 ± 5.32
	20.50	90.21 ± 5.73	5.93	87.32 ± 6.11	6.32	88.14 ± 6.01
	41.00	92.01 ± 4.21	4.55	90.00 ± 7.25	9.62	91.39 ± 3.65
NOL	50.32	90.36 ± 3.55	3.71	89.32 ± 8.26	8.88	80.69 ± 3.01
	100.65	91.30 ± 2.89	3.21	90.36 ± 7.69	7.86	84.32 ± 2.95
	201.30	89.52 ± 3.40	3.66	87.09 ± 4.14	4.33	85.24 ± 4.14
ZL	180.84	86.33 ± 6.55	7.32	86.48 ± 6.55	7.33	85.55 ± 4.95
	361.69	85.99 ± 3.60	3.88	86.44 ± 6.47	6.69	82.36 ± 5.22
	723.38	90.78 ± 4.87	5.09	87.05 ± 4.89	5.38	86.77 ± 4.44
BP	11.39	86.57 ± 6.28	8.15	87.19 ± 8.47	10.32	77.88 ± 8.88
	22.78	85.99 ± 6.02	6.25	85.84 ± 8.49	9.14	75.36 ± 7.62
	45.55	87.64 ± 6.30	6.74	86.28 ± 9.01	8.96	79.32 ± 5.36

### Pharmacokinetic study

**Fig. 8:**
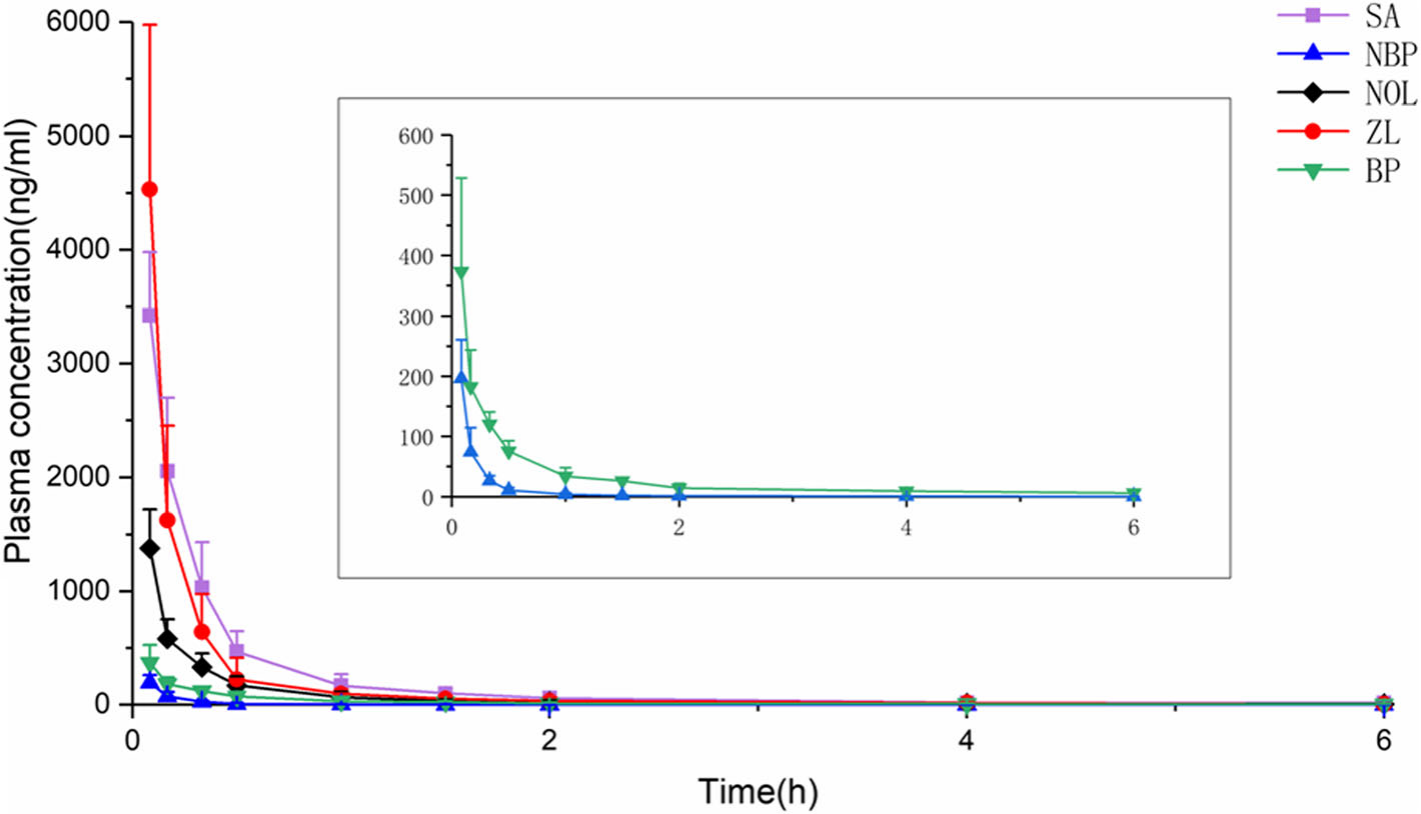
Time-plasma concentration curve of VOC by intravenous administration in rats. Values represent the means ± standard error (*n* = 6)

Pharmacokinetic parameters of VOC by oral and intravenous administration were listed in Table 5, and the mean plasma concentration–time profiles of the analytes were displayed in Fig. 8. On the basis of the biopharmaceutics classification system (BCS), volatile compounds belong to class 2, with low solubility and high permeability, which mainly undergo passive transport across plasma membranes [18, 19]. The absorption of SA, NBP, NOL, ZL and BP were rapid, with peak concentrations occurring before 30 min after oral administration. Among them, the T_max_ of SA, NOL and ZL were about 12 min, which meant they absorbed more quickly than NBP and BP [32, 33]. Compared with intravenous injection, its peak concentration was lower, and the absolute bioavailability of the five components was less than 25%, which was partly because of extensive first-pass metabolism in the liver [39, 40]. SA was metabolically instability in hepatocytes, and hydroxylation, epoxidation, aromatization and GSH conjugation were the main metabolic pathways [43]. NBP was well absorbed and extensively metabolized by multiple enzymes to various metabolites prior to urinary excretion [5, 6]. The low oral bioavailability may because the largescale metabolic decomposition of ZL in the liver and intestinal metabolic system, and rapidly entered the central nervous system [38].

**Table 5:** Pharmacokinetic parameters of VOC by oral and intravenous administration. Values represent the means ± standard error (*n* = 6)

components	Group	C_max_ (μg/l)	T_max_ (h)	t_1/2α_ (h)	t_1/2β_ (h)	MRT_0-t_ (h)	AUC_0-t_ (μg/l*h)	*F*_1_(%)
SA	iv	3425.09 ± 556.79	0.083 ± 0.00	0.09 ± 0.05	0.78 ± 0.66	0.72 ± 0.08	1370.79 ± 314.70	/
	po	630.64 ± 309.58	0.19 ± 0.10	1.31 ± 1.58	3.79 ± 1.40	4.19 ± 0.49	1260.98 ± 273.94	22.30
NBP	iv	196.44 ± 64.48	0.083 ± 0.00	0.06 ± 0.04	0.26 ± 0.15	0.54 ± 0.22	54.64 ± 16.50	/
	po	25.94 ± 8.30	0.47 ± 0.07	0.26 ± 0.10	0.65 ± 0.74	2.70 ± 0.47	36.11 ± 7.58	16.32
NOL	iv	1381.81 ± 334.90	0.083 ± 0.00	0.04 ± 0.04	0.30 ± 0.11	0.79 ± 0.17	504.65 ± 152.08	/
	po	222.78 ± 71.31	0.18 ± 0.12	0.46 ± 0.67	3.10 ± 2.18	3.42 ± 0.57	442.24 ± 45.19	21.90
ZL	iv	4531.32 ± 1447.37	0.083 ± 0.00	0.04 ± 0.04	0.55 ± 0.66	0.45 ± 0.10	1239.94 ± 434.83	/
	po	213.68 ± 76.01	0.22 ± 0.09	0.82 ± 0.51	5.48 ± 2.61	3.94 ± 0.54	503.96 ± 53.33	10.16
BP	iv	373.30 ± 155.03	0.083 ± 0.00	0.04 ± 0.04	0.70 ± 0.54	1.28 ± 0.32	198.46 ± 42.81	/
	po	43.70 ± 10.89	0.50 ± 0.28	1.38 ± 1.02	3.74 ± 3.26	3.46 ± 1.08	99.09 ± 23.33	12.43

**Fig. 9:**
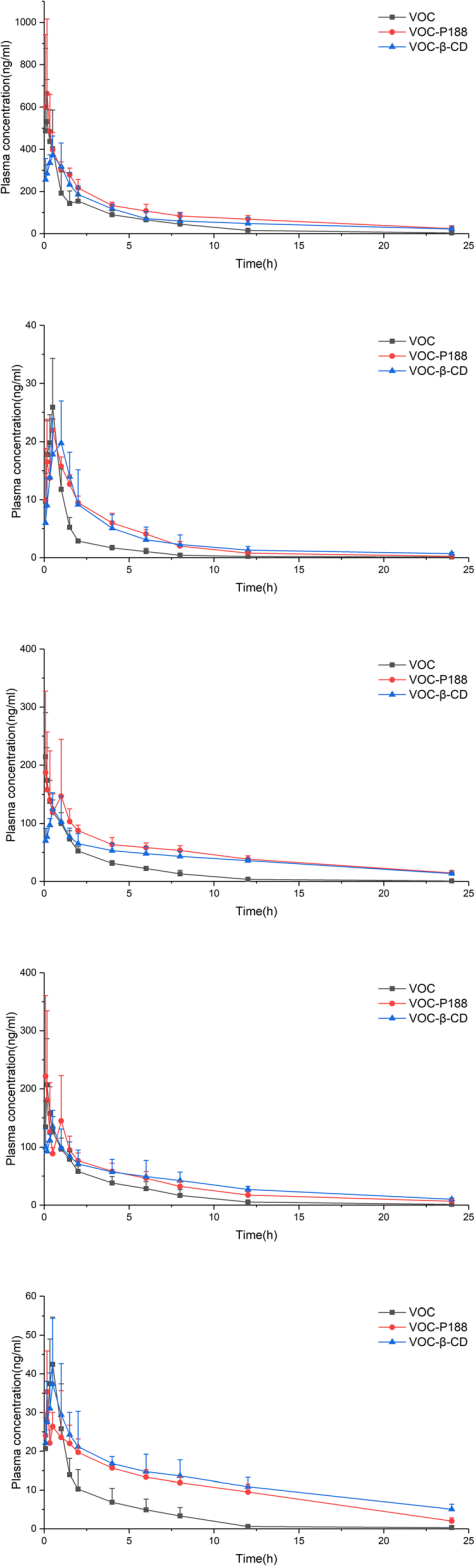
Time-plasma concentration curve of VOC, VOC-P188 and VOC-β-CD by oral administration in rats. Values represent the means ± standard error (*n* = 6)

**Fig. 10:**
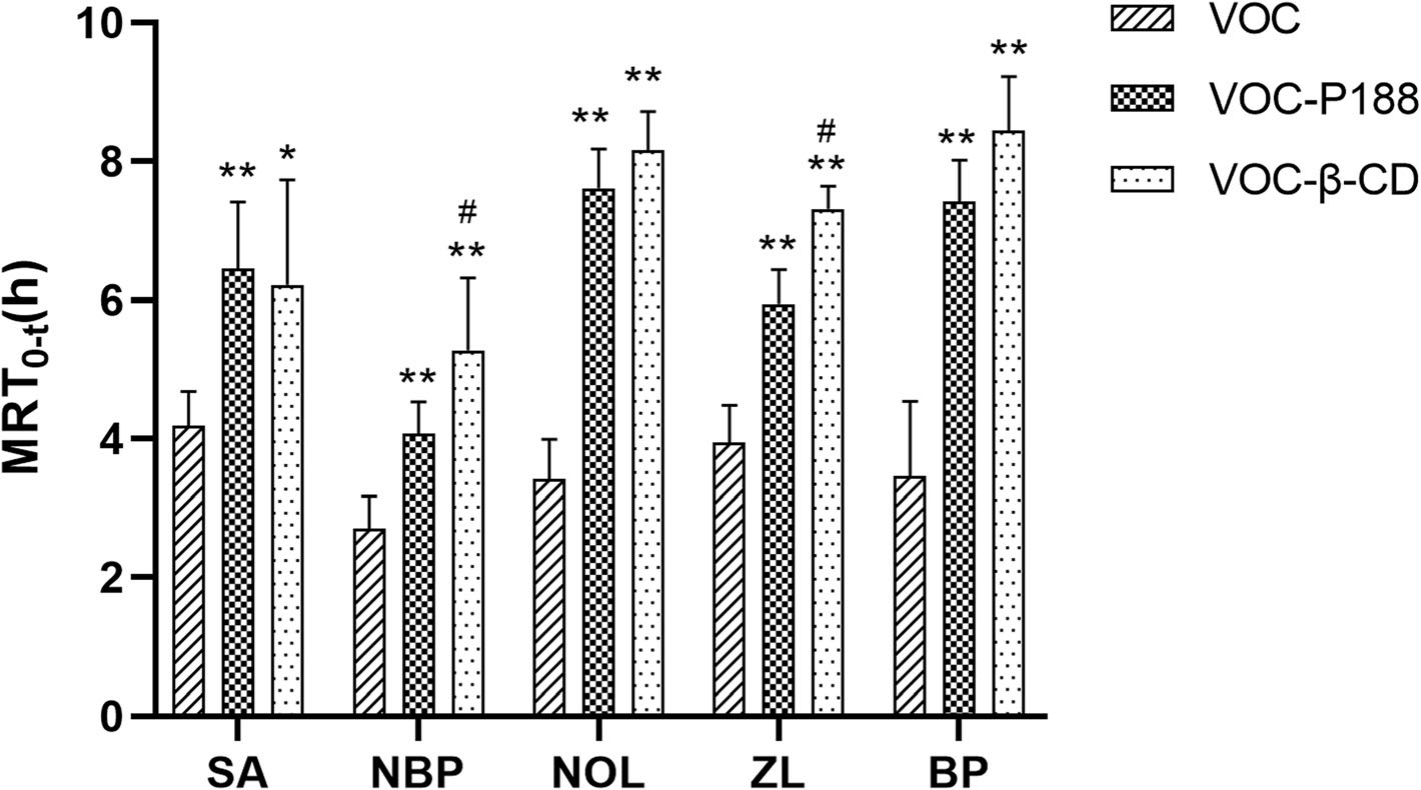
Mean residence time (MRT) of VOC, VOC-P188 and VOC-β-CD. Values represent the means±standard error (*n* = 6). * *p* < 0.05, vs VOC; **p < 0.01, vs VOC; ^#^*p* < 0.05, vs VOC-P188

Pharmacokinetic parameters of VOC-P188 and VOC-β-CD by oral administration were listed in Table 6 [47], and the mean plasma concentration-time profiles of the analytes were displayed in Fig. 9. The mean residence time (MRT) of VOC, VOC-P188 and VOC-β-CD were shown in Fig. 10. The process of each component in rats conformed to the two-compartment model after oral administration of VOC, VOC-P188 and VOC-β-CD. After VOC was prepared into new formulations by solid dispersion technology and inclusion technology, MRT of five components was significantly prolonged when compared with VOC, which indicated that the preparations were more conducive to maintain drug concentration in the plasma [13]. Compared with VOC-P188, MRT of NBP and ZL in VOC-β-CD was significantly extended (*p* < 0.05). The reason may that VOC was highly dispersed in P188, but VOC was encapsulated in the β-CD molecular cavity and it took some time for VOC to be released from β-CD molecular cavity.

**Table 6:** Pharmacokinetic parameters of VOC-P188 solid dispersion and VOC-β-CD inclusion by oral administration. Values represent the means ± standard error (*n* = 6)

components	Group	C_max_ (μg/l)	T_max_ (h)	t_1/2α_ (h)	t_1/2β_ (h)	MRT_0-t_ (h)	AUC_0-t_ (μg/l*h)	*F*_2_(%)
SA	VOC	630.64 ± 309.58	0.19 ± 0.10	1.31 ± 1.58	3.79 ± 1.40	4.19 ± 0.49	1260.98 ± 273.94	*/*
	VOC-P188	700.75 ± 348.70	0.20 ± 0.16	1.50 ± 1.59	7.24 ± 2.57	6.45 ± 0.96^**^	2363.17 ± 350.93^**^	185.82
	VOC-β-CD	398.92 ± 68.35^#^	0.50 ± 0.26^*#^	2.34 ± 1.24	2.98 ± 0.57	6.22 ± 1.51^*^	1825.75 ± 532.32^#^	138.69
NBP	VOC	25.94 ± 8.30	0.47 ± 0.07	0.26 ± 0.10	0.65 ± 0.74	2.70 ± 0.47	36.11 ± 7.58	/
	VOC-P188	23.48 ± 1.59	0.32 ± 0.20	2.82 ± 1.55^**^	3.13 ± 1.01^*^	4.07 ± 0.46^**^	72.70 ± 9.80^**^	197.67
	VOC-β-CD	22.42 ± 5.56	0.83 ± 0.26^**##^	3.95 ± 1.96^**^	4.39 ± 1.82^**^	5.28 ± 1.04^**##^	76.06 ± 24.65^**^	198.39
NOL	VOC	222.78 ± 71.31	0.18 ± 0.12	0.46 ± 0.67	3.10 ± 2.18	3.42 ± 0.57	442.24 ± 45.19	/
	VOC-P188	243.68 ± 130.94	0.42 ± 0.45	4.21 ± 4.29^*^	8.84 ± 1.84^**^	7.61 ± 0.57^**^	1138.94 ± 63.85^**^	258.18
	VOC-β-CD	134.07 ± 24.28^#^	0.67 ± 0.26^*^	3.21 ± 2.44	9.77 ± 2.71^**^	8.17 ± 0.55^**^	944.58 ± 84.96^**##^	218.01
ZL	VOC	213.68 ± 76.01	0.22 ± 0.09	0.82 ± 0.51	5.48 ± 2.61	3.94 ± 0.54	503.96 ± 53.33	/
	VOC-P188	319.61 ± 285.56	0.49 ± 0.42	4.40 ± 4.10^*^	8.91 ± 5.25	5.95 ± 0.49^**^	801.47 ± 199.45^**^	163.35
	VOC-β-CD	145.01 ± 24.76	0.40 ± 0.17	4.72 ± 1.92^*^	5.96 ± 2.90	7.31 ± 0.33^**##^	883.20 ± 210.53^**^	224.54
BP	VOC	43.70 ± 10.89	0.50 ± 0.28	1.38 ± 1.02	3.74 ± 3.26	3.46 ± 1.08	99.09 ± 23.33	/
	VOC-P188	40.26 ± 8.37	0.42 ± 0.33	2.72 ± 3.66	8.64 ± 2.44^*^	7.42 ± 0.60^**^	249.21 ± 24.50^**^	251.50
	VOC-β-CD	38.80 ± 15.96	0.35 ± 0.19	7.02 ± 5.42^*^	19.57 ± 5.63^**##^	8.45 ± 0.77^**^	298.49 ± 31.68^**##^	363.55

* *p* < 0.05, vs VOC; **p < 0.01, vs VOC; ^#^*p* < 0.05, vs VOC-P188; ^##^*p* < 0.01, vs VOC-P188

Except for BP, the *T*max value of SA, NBP, NOL and ZL in VOC-P188 and VOC-β-CD was prolonged. The formulation of VOC-P188 and VOC-β-CD could slow down the absorption process of the analytes. The Cmax value of the five components in VOC-β-CD was lower, when compared with VOC, which indicated that this formulation made the components release more smoothly in vivo. Both VOC-P188 and VOC-β-CD had a significantly higher AUC in comparison with VOC, and gave between 1.3-fold to 3.6-fold improved oral bioavailability of five compounds in VOC (Table 6). The inclusion compound showed higher AUC of BP than did the solid dispersion (*p* < 0.01), while the solid dispersion showed higher AUC of SA and NOL than did the inclusion compound (*p* < 0.05, *p* < 0.01).

In addition, the stability test results showed that the content of SA and ZL in VOC-P188 was decreased by 15.8 and 38.9% respectively at 60 °C for 10 days, and the content of SA and ZL in VOC-β-CD was decreased by 9.0 and 20.3% respectively at the same temperature. Compared with VOC, the two preparations reduced the active ingredient content to a lesser extent. Humidity had a great influence on the stability of VOC in the two preparations. The contents of SA, NBP, NOL, ZL and BP decreased in different degrees at 92.5% relative humidity for 10 days. Furthermore, the contents of the five phthalides in the two preparations were decreased by less than 10% under the illumination condition (4500 lx ± 500 lx). In general, the five phthalides in the inclusion compound were more stable than in the solid dispersion. VOC-β-CD had a white appearance and was similar in color to β-CD, which indicated that VOC have been encapsulated in the cavity of β-CD. More importantly, VOC was brownish yellow oil and had extremely pungent smell, while VOC-β-CD had almost no pungent smell for people in our lab. Literatures showed that the inclusion complex not only effectively enhanced the dissolution of drugs in aqueous solutions, but also showed the potential to mask its bitter taste in the oral cavity [4, 29, 30, 42, 48]. VOC-P188 had a faint yellow appearance and still had pungent smell.

In fact, both the bioavailability of SA, NBP, NOL, ZL and BP in the two preparations was still less than 50%, although the relative bioavailability and the stability had been significantly improved. Other new dosage forms such as microcapsule [44], micelles [28] and solid lipid nanoparticles [45] could be used to make the phthalides to be released more slowly in vivo and improve oral bioavailability.

## Conclusion

SA, NBP, NOL, ZL and BP in VOC from chaxiong possessed poor absolute oral bioavailability, which was less than 25%. In this paper, VOC were prepared as VOC-P188 solid dispersions and VOC-β-CD inclusion compound, and the physicochemical property which was done with FT-IR, TEM, DSC, PXRD, indicated that these two formulations were successfully formed. Furthermore, both VOC-P188 solid dispersions and VOC-β-CD inclusion compound significantly increased the dissolution in vitro and improved oral bioavailability of these five phthalides in vivo when compared with VOC. However, the bioavailability of SA, NBP, NOL, ZL and BP in the two preparations was still less than 50%, other new dosage forms could be used to further improve the bioavailability.

## Data Availability

All data and materials are available and could be obtained from the corresponding author Zhang G.S.

## Abbreviations

VOC: Volatile oil of chaxiong
SA: Senkyunolide A
NBP: N-butylphthalide
NOL: Neocnidilide
ZL: Z-ligustilide
BP: Butenyl phthalide
P188: Poloxamer 188
β-CD: β-cyclodextrin
DL: Dehydrocostus lactone
VOC-P188: VOC-P188 solid dispersion
VOC-β-CD: VOC-β-CD inclusion compound
SEM: Scanning electron microscopy
FT-IR: Fourier transform infrared spectroscopy
DSC: Differential scanning calorimetry
PXRD: X-ray powder diffraction
PBS: Phosphate buffer solution
LC–MS/MS: Liquid chromatograph-mass spectrometer
HPLC: High performance liquid chromatography
IS: Internal standard
Iv: Intravenous injection
Po: Peros
Tmax: Time to maximum concentration
Cmax: Maximum concentration
t_1/2α_: Distributed half-life
t_1/2β_: Elimination half life
MRT: Mean retention time
AUC: Area under the curve
CL: Clearance
HPLC: High Performance Liquid Chromatography
SD rats: Sprague Dawley rats
EP: Heparinized Eppendorf
QC: Quality control
SD: Square deviation
LLOQ: Lower limit of quantification
RSD: Rlative Sandard Deviation
GSH: Glutathione
BCS: Biopharmaceutics classification system
